# Ovarian cyst regression with levothyroxine in ovarian hyperstimulation syndrome associated with hypothyroidism

**DOI:** 10.1530/EDM-13-0006

**Published:** 2013-07-01

**Authors:** Roghieh Molaei Langroudi, Fatemeh Ghazanfari Amlashi, Mohammad Hassan Hedayati Emami

**Affiliations:** Diagnostic Radiology DepartmentPoursina Hospital, Guilan University of Medical SciencesGuilanIran; 1 Guilan Endocrinology and Metabolism Research Center, Razi HospitalRasht, GuilanIran; 2 Department of EndocrinologyGuilan Endocrinology and Metabolism Research Center, Razi HospitalRasht, GuilanIran

## Abstract

Background: Spontaneous ovarian hyperstimulation syndrome (sOHSS) can occur following hypothyroidism. Ultrasonography facilitates diagnosis and monitoring of this syndrome. We describe ovarian sonographic changes in a hypothyroid patient with sOHSS after treatment with levothyroxine (l-T_4_).

Case presentation: A 15-year-old girl presented with abdominal pain and distension for a few months. On examination, she had classical features of hypothyroidism. Abdominal and pelvic ultrasound revealed enlarged ovaries with multiple thin-walled cysts and mild ascitic fluid. On follow-up, abdominal ultrasound showed significant reduction of ovary size after 6 weeks of initiation of l-T_4_. Normal ovary size with complete regression of ovarian cysts was seen after 4 months.

Conclusion: Serial ultrasound in sOHSS associated with hypothyroidism showed regression of ovarian cysts and ovarian volume after 4 months whereas in other studies, it is reported to happen in various durations, presumably according to its etiology.

**Learning points:**

- OHSS can rarely occur due to hypothyroidism.
- This type of OHSS can be simply treated by l-T_4_ replacement, rather than conservative management or surgery in severe cases.
- Ultrasound follow-up shows significant regression of ovarian size and cysts within 6 weeks of initiation of l-T_4_.
- Ultrasound follow-up shows normal ovarian size with complete resolution of ovarian cysts 4 months after treatment.

## Background

Ovarian hyperstimulation syndrome (OHSS) is usually iatrogenic and is a potentially life-threatening complication of ovulation induction. Spontaneous OHSS might occur following high levels of human chorionic gonadotropin (HCG) in normal pregnancy, hypothyroidism, or FSH receptor mutation (1). Expanding use of ultrasonography facilitates the diagnosis and monitoring of the treatment of this syndrome (2).

We have described this syndrome in a girl virgin with primary autoimmune hypothyroidism in our previous article (3); we followed her by serial abdominal ultrasound that showed normal ovary size and regression of ovarian cysts after levothyroxine (l-T_4_) replacement.

## Case presentation

A 15-year-old girl presented with abdominal pain and distension for a few months. On examination, she had classical features of hypothyroidism (3). The abdomen was distended and non-tender with a large palpable mass in the lower abdomen extending to the upper abdomen.

## Investigation

Laboratory findings included the following: Hb=11.2 g/dl, Hct=36.2%, MCV=81 fl, MCH=28.2 pg, BUN=13 mg/dl, Cr=0.7 mg/dl, cholesterol=290 mg/dl, and TG=273 mg/dl. Hormonal studies confirmed hypothyroidism: serum TSH >100 mIU/l, total T_4_=1.8 μg/dl (normal: 4.4–12.5 μg/dl, radioimmunoassay (RIA)), T3RU=31.2% (normal: 25–34.4%), anti-TPO antibody=290 U/ml (normal <70, ELISA), and prolactin=176 ng/ml (normal: 3–21, RIA) (3). Abdominal and pelvic ultrasound revealed enlarged ovaries that occupied the whole abdomen and pelvic cavity: right ovary, 150×75×62 mm with a volume of 454 cc; left ovary, 130×70×68 mm with a volume of 340 cc. It also represented multiple thin-walled cysts and mild ascitic fluid. Abdominal and pelvic computed tomography (CT) scan showed these thin-walled cysts with no enhancement.

## Treatment

She was started on l-T_4_ 100 μg/day.

## Outcome and follow-up

**Figure 1:**
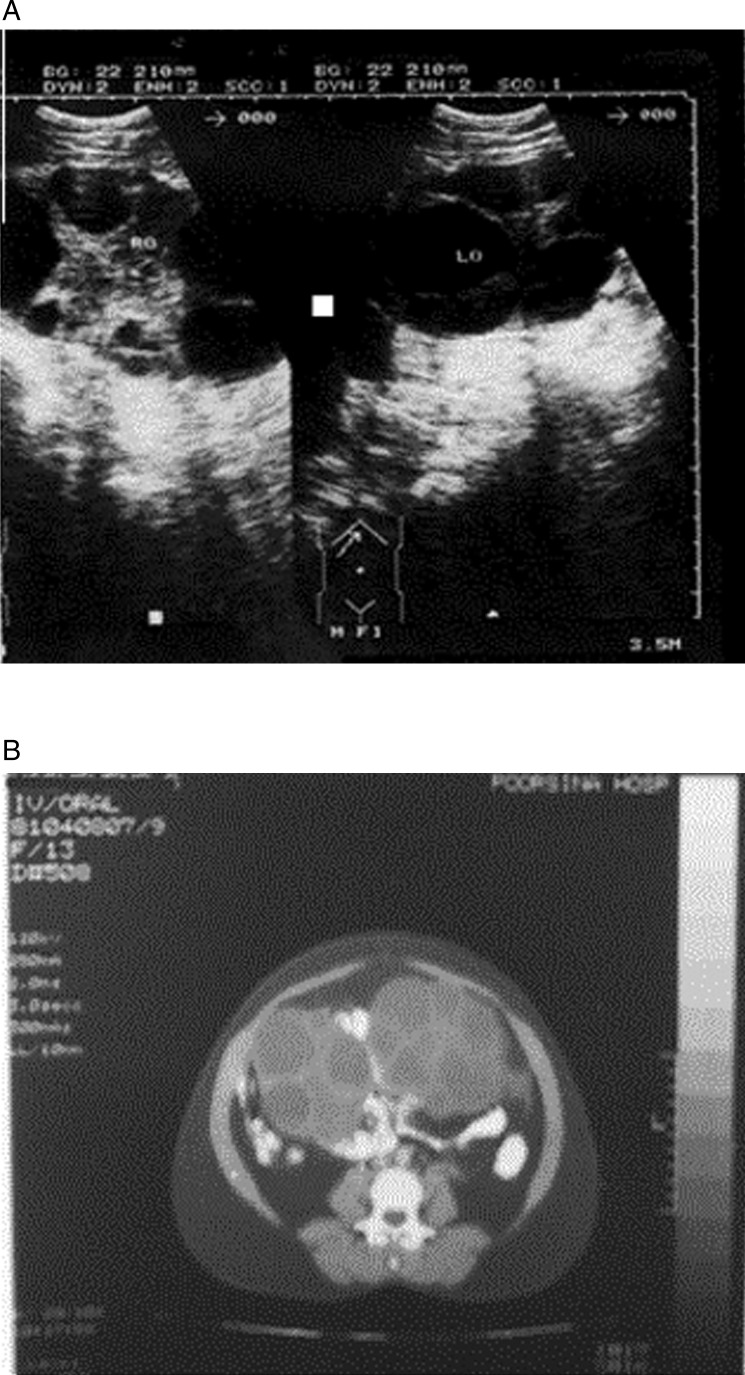
Imaging findings show bilateral multilobulated ovarian cysts. (A) Abdominal ultrasound and (B) abdominal CT scan (3).

**Figure 2:**
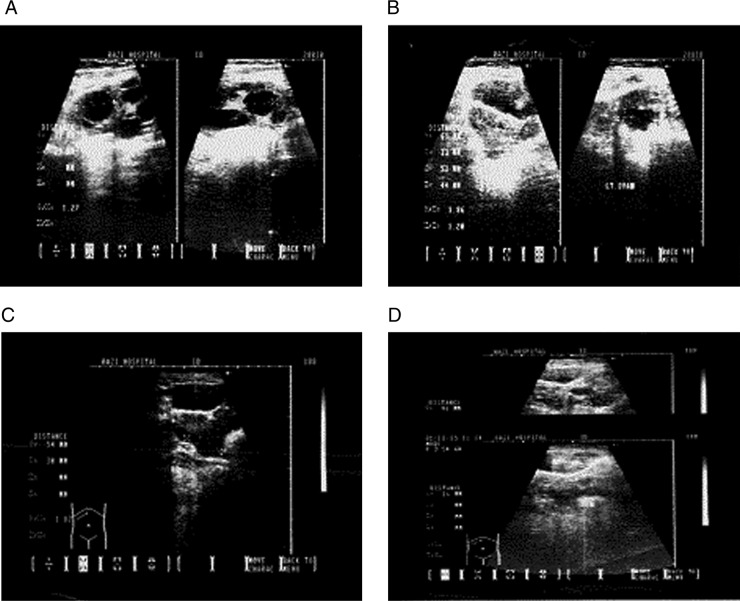
Follow-up abdominal sonography showed significant ovarian volume and cyst regression within 4 months of levothyroxine therapy (A) after 2 months, (B) after 4 months, (C) after 8 months (right ovary), and (D) after 8 months (left ovary).

On follow-up ultrasound, the size of the ovaries became significantly smaller 6 weeks after l-T_4_ replacement and became normal with complete resolution of cysts after 4 months (Figs 1 and 2).

## Discussion

A description of OHSS in two members of a family has recently been published (3), but there are a few studies focusing on ovarian volume and cyst regression after l-T_4_ replacement therapy. Imaging findings in OHSS include multiple, large, and thin-walled cysts and ascitic fluid in severe forms (4). The exclusion of diagnosis of ovarian cancer is made by ultrasonography and CT scan or magnetic resonance imaging (MRI), which reveals the classical ‘spoke wheel’ appearance that is characteristic of theca lutein cysts without solid components. Furthermore, the reduction in ovarian volume and regression of detected cysts during close observational management and ultrasonic follow-up can differentiate OHSS from other diagnoses (5).

**Figure 3:**
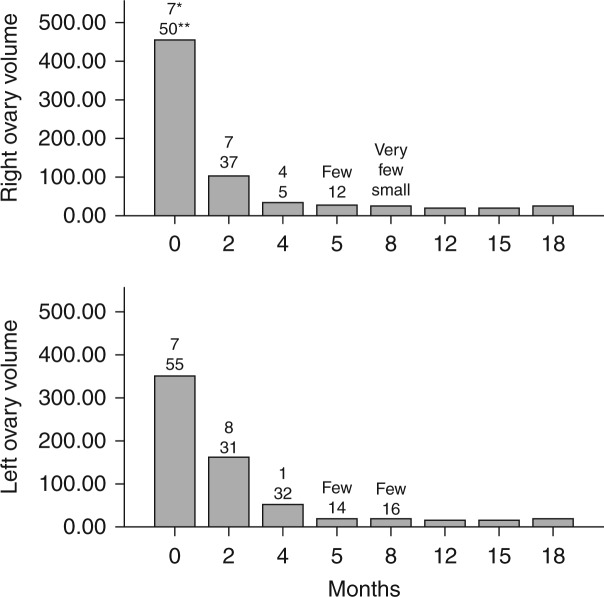
Right and left ovarian volume change after levothyroxine replacement. *Upper digit shows the number of cysts and **lower digit shows the largest diameter of the largest cyst.

Here, we described resolution of ovarian cysts and normalization of the size of the ovaries in our patient 4 months after l-T_4_ administration (Fig. 3). It is noteworthy that the kinetics of the symptoms are closely related to the life span of corpus luteum. In the absence of pregnancy, symptoms resolve spontaneously with the onset of menses, while in the presence of pregnancy, symptoms start to improve after the sixth week of pregnancy, before HCG peak (1). However, in OHSS with underlying disease such as hypothyroidism, complicated pregnancies or in the presence of mutated FSH receptor genes, the symptoms have been reported to last longer (6) (7) (8) (9) (10) (11) (12) (13) (14). Mousavi *et al*. (6) reported normalization of ovarian appearance in ultrasound 6 months after l-T_4_ replacement therapy. In other studies on hypothyroid patients (with and without pregnancy), considerable regression of cysts was observed after 3 months (7) (8) (9) (10) (11), with an exception that in three case reports patients experienced total regression 3 months after delivery (12) (13) (14) (Table 1). Rising serum level of endogenous HCG might strengthen the severity of OHSS in pregnant patients and would lead to a more complicated course than patients with hypothyroidism (15).

**Table 1:** Summary of case reports describing patients with OHSS associated with hypothyroidism

**Reference**	**Age** (years)	**Hypothyroidism**	**Pregnancy**	**FSH receptor mutation**	**Sonographic report**	**Treatment**	**Follow-up**
Hedayati *et al*. (3)	15	TSH >100 mIU/l Ab:Neg.	–	Neg.	Enlarged ovaries with multiple ovarian cysts Rt: 150×75×62 Lt: 130×70×68	Levothyroxine (100 μg)	After 4 months: normal ovary size and regression of cysts
	14.5	TSH=72.5 mIU/l	–	NA	Multiple large cysts with rupture of one cyst Rt: 110×65 Lt: 118×58	Levothyroxine (100 μg)		After 4 months: normal
Akbay *et al*. (13)	21 (P1)	TSH=8.75 mIU/l Ab:NA	10 weeks HCG=Nl	NA	Bilateral multilobulated cystic 130×80 sized ovaries	Levothyroxine (100 μg)	After 3 months of delivery: normal
	23 (P2)	TSH=2.16 mIU/l Ab:NA	12 weeks HCG=Nl		Bilateral multilobulated cysts Rt: 13×70 Lt: 110×70	Levothyroxine (100 μg)	After 2 months of delivery: normal
Dietrich *et al*. (15)	26 (P1)	Normal	12 weeks HCG=118 665	Present (D567N)	Bilateral multicystic ovaries Rt: 140×150 Lt: 120×130	Conservative	Abortion at 15 weeks
					Enlarged ovaries		
	26 (P2)	TSH=5.51 mIU/l Ab:NA	10 weeks HCG=147 688		Rt: 100×100 Lt: 100×100	Levothyroxine (100 μg)	Normal delivery at term
Lussiana *et al*. (16)	29	TSH=5.92 mIU/l Ab:NA	22 weeks (with abortion)	Present (with undetermined significance)	Bilateral multiple ovarian cysts Rt: 200×110 Lt: 160×120	Levothyroxine	After 3 months of abortion: normal ovaries
Edwards *et al*. (9)	30	TSH=41.7 mIU/l	10 weeks HCG=291 206	NA	Enlarged mass	Levothyroxine	By 22 weeks of gestation: ovarian regression
Borna *et al*. (12)	30	TSH >400 mIU/l Ab:NA	20 weeks HCG=Nl	NA	Bilateral multilobulated ovarian cysts Rt: 200×160 Lt: 160×100	Levothyroxine (200 μg)	10 weeks after delivery: normal ovaries
Sultan *et al*. (11)	12	TSH=1310 mIU/l Ab:Neg.	–	Neg.	Large cystic structure	Levothyroxine	After 3 months: resolution of cysts
Mousavi *et al*. (6)	26	TSH >50 mIU/l Ab:NA	–	NA	Bilateral multiseptated ovarian masses Rt: 69×63×96 Lt: 66×63×99	Levothyroxine (100 μg)	After 6 months: normal ovary size
Taher *et al*. (10)	22	TSH >100 mIU/l Ab:NA	–	NA	Bilateral multilobulated ovarian mass with cystic component Rt: 90×120 Lt: 60×40	Levothyroxine (100 μg)	After 3 months: marked reduction
Corsado *et al*. (7)	25	TSH=210 mIU/l Ab:Neg.	11 weeks HCG=15 890	NA	Bilateral multilobulated ovarian cysts Rt: 160×150 Lt: 160×130	Levothyroxine (100 μg)	By 24 weeks of gestation: normal ovary

Ab, antithyroglobulin/antiperoxidase antibody; Neg, negative; NA, not available; P, pregnancy; HCG, human chorionic gonadotropin (IU/l); Nl, normal (according to gestational age); Rt and Lt, right and left ovaries (mm^2^).

In conclusion, ultrasonography as well as CT scan or MRI assists the diagnosis of OHSS. By serial ultrasound, we observed regression of ovarian cysts and ovarian volume after 4 months whereas in other studies, it is reported to happen in various durations that may be related to the etiology of this syndrome.

## References

[1] Debaere A , Smits G , De Leener A , Costagliola S & Vassart G . 2005 Understanding ovarian hyperstimulation syndrome. Endocrine. 26: 285–289 10.1385/ENDO:26:3:285 16034183

[2] McNeary M & Stark P . 2002 Radiographic findings in ovarian hyperstimulation syndrome. Journal of Thoracic Imaging. 17: 230–232 10.1097/00005382-200207000-00009 12082376

[3] Hedayati Emami MH , Molaei Langroudi R & Ghazanfari Amlashi F . 2012 Ovarian hyperstimulation syndrome and autoimmune primary hypothyroidism in two members of a family. Journal of Clinical Case Reports. 2: 113 10.4172/2165-7920.1000113

[4] Salem SH. Abdominal, pelvic, thoracic sonography: gynecology. In *Diagnostic Ultrasound*, 4th edn, ch 15, pp 575–576. Eds CM Rumack, SR Wilson, JW Charboneau & D Levine. Philadelphia: Elsevier Mosby, 2011.

[5] Haimov-Kochman R , Yanai N , Yagel S , Amsalem H , Lavy Y & Hurwitz A . 2004 Spontaneous ovarian hyperstimulation syndrome and hyperreactio luteinalis are entities in continuum. Ultrasound in Obstetrics & Gynecology. 24: 675–678 10.1002/uog.1759 15476296

[6] Mousavi AS , Behtash N , Hasanzadeh M , Modares Gilani M , Ghaemmaghami F , Shahroch E & Nejad T . 2005 Spontaneous ovarian hyperstimulation syndrome caused by hypothyroidism. Cancer Therapy. 3: 397–400

[7] Cordaso C , Olode N & Soares L . 1999 Spontaneous ovarian hyperstimulation and primary hypothyroidism with a naturally conceived pregnancy. Obstetrics and Gynecology. 39: e64–e67 10.1016/s0029-7844(98)00435-9 10912402

[8] Nappi RG , Di Naro E , D'Aries AP & Nappi L . 1998 Natural pregnancy in hypothyroid woman complicated by spontaneous ovarian hyperstimulation. American Journal of Obstetrics and Gynecology. 178: 610–611 10.1016/S0002-9378(98)70448-X 9539535

[9] Edwards-silva RN , Han CS , Hoang Y & Kao LC . 2008 Spontaneous ovarian hyperstimulation in a naturally conceived pregnancy with uncontrolled hypothyroidism. Obstetrics and Gynecology. 111: 498–501 10.1097/01.AOG.0000279139.12412.90 18238999

[10] Taher BM , Ghariabeh RA & Jarrah NS . 2004 Spontaneous ovarian hyperstimulation syndrome caused by hypothyroidism in an adult. European Journal of Obstetrics, Gynecology, and Reproductive Biology. 112: 107–109 10.1016/S0301-2115(03)00283-5 14687752

[11] Sultan A , Velaga MR , Fleet M & Cheetham T . 2006 Cullen's sign and massive ovarian enlargement secondary to primary hypothyroidism in a patient with a normal FSH receptor. Archives of Disease in Childhood. 91: 509–510 10.1136/adc.2005.088443 16714722 PMC2082807

[12] Borna S & Nasery A . 2007 Spontaneous ovarian hyperstimulation in a pregnant woman with hypothyroidism. Fertility and Sterility. 88: 705.e1–705.e3 10.1016/j.fertnstert.2006.12.003 17433320

[13] Akbay E , Uzunçakmak C , Sevda Idil N , Akçiğ Z , Özel G & Yaşar L . 2010 Recurrent spontaneous ovarian hyperstimulation syndrome with hypothyroidism: a case report. Medical Journal of Bakirköy. 6: 42–45

[14] Michaelson-Cohen R , Altarescu G , Beller U , Reens R , Halevy-Shalem T & Eldar-Geva T . 2008 Does elevated human chorionic gonadotropin alone trigger spontaneous ovarian hyperstimulation syndrome?. Fertility and Sterility. 90: 1869–1874 10.1016/j.fertnstert.2007.09.049 18166181

[15] Dietrich M , Bolz M , Reimer T , Costagliola S & Gerber B . 2010 Two different entities of spontaneous ovarian hyperstimulation in a woman with FSH receptor mutation. Reproductive Biomedicine Online. 20: 751–758 10.1016/j.rbmo.2010.02.017 20378412

[16] Lussiana C , Guani B , Restagno G , Rovei V , Menato G , Revelli A & Massobrio M . 2009 Ovarian hyper-stimulation syndrome after spontaneous conception. Gynecological Endocrinology. 25: 455–459 10.1080/09513590902898213 19499413

